# Synthesis of Thermoplastic Xylan-Lactide Copolymer with Amidine-Mediated Organocatalyst in Ionic Liquid

**DOI:** 10.1038/s41598-017-00464-6

**Published:** 2017-04-03

**Authors:** Xueqin Zhang, Huihui Wang, Chuanfu Liu, Aiping Zhang, Junli Ren

**Affiliations:** 1 State Key Laboratory of Pulp and Paper Engineering, South China University of Technology, Guangzhou, 510640 China; 2 College of Materials and Energy, Guangdong Key Laboratory for Innovative Development and Utilization of Forest Plant Germplasm, South China Agricultural University, Guangzhou, 510642 P.R. China

## Abstract

Ring-opening graft polymerization (ROGP) of l-Lactide (l-LA) is a practical method of altering the physical and chemical properties of lignocellulose. Previous studies have mainly investigated cellulose and tin-based catalysts, particularly of tin(II) 2-ethylhexanoate (Sn(oct)_2_), at high temperatures and reported low graft efficiencies. In the present study, ROGP of l-LA was successfully achieved on xylan-type hemicelluloses in ionic liquid (IL) 1-allyl-3-methylimidazolium chloride ([Amim]Cl) using 1,8-diazabicyclo[5.4.0]undec-7-ene (DBU) as an effective organic catalyst. Mild reaction condition (50 °C) was used to limit transesterification, and thus enhance the graft efficiency. The hydroxyl groups on xylan acted as initiators in the polymerization, and DBU, enhanced the nucleophilicity of the initiator and the propagating chain. Xylan-*graft*-poly(l-Lactide) (xylan-*g*-PLA) copolymer with a degree of substitution (DS) of 0.58 and a degree of polymerization (DP) of 5.51 was obtained. In addition, the structures of the xylan-*g*-PLA copolymers were characterized by GPC, FT-IR and NMR, confirming the success of the ROGP reaction. Thermal analysis revealed that the copolymers exhibited a single glass-transition temperature (*T*_g_), which decreased with increasing molar substitution (MS). Thus, modification resulted in the graft copolymers with thermoplastic behavior and tunable *T*_g_.

## Introduction

Hemicelluloses, the major non-cellulose polysaccharides in wood component, are a renewable and biodegradable resource, representing about 20–40% of the biomass of plants^1, 2^. Hemicelluloses contain varieties of sugar units, primarily xylans, mannans, glucans and xyloglucans, in various proportions depending on the natural source^3, 4^. Xylans are the most abundant hemicelluloses^5^. Recently, the production of novel composite polymers from hemicelluloses has received increasing interests due to the depletion of non-biodegradable fossil resources^1, 6–8^. However, for some applications, the properties like solubility, hydrophobicity and compatibility of hemicellulose-based polymers should be improved or tailored. Chemical modifications have been applied to confer desirable properties and functionalities to hemicelluloses, thus resulting in the improved performance of composite materials^9–12^, among which graft polymerization provides a significant route to covalently modify the surface of hemicelluloses with polymers^4, 13–15^.

Aliphatic polyesters, with desirable biocompatibility, biodegradability and permeability, are potential candidates as matrixes in biocomposites^16–19^. Poly(l-Lactide) (PLA), one of the most promising and practical biopolymers, is a hydrophobic and thermoplastic biopolymer that can be derived from renewable sources (mainly starch and sugar)^16, 20–23^. PLA is a candidate for applications in various fields, including biomedical products, food packaging, fibre production and films for agro-industry^24–27^. Compared to traditional oil-based polymers, PLA exhibits high strength and stiffness and desirable optical, physical and mechanical properties^28–30^. However, the high cost of production of PLA limits its ability to directly replace conventional synthetic polymers^31^.

Recent advances in polymerization techniques have enabled more economical production of PLA and have broadened its applications^28, 32, 33^. Among them, ring-opening graft polymerization (ROGP) of l-LA onto renewable lignocellulose represents a practical means of altering the chemical properties of lignocellulose, and grafting PLA onto cellulose or cellulose derivatives have attracted considerable research interests^15, 32, 34^. Traditionally, metal-based catalysts like tin(II) 2-ethylhexanoate (Sn(oct)_2_) are used to catalyze grafting modification of cellulose with polyesters. For example, Guo *et al.* ^35^ and Dong *et al*.^23^ synthesized cellulose-*graft*-poly(l-Lactide) copolymers using a Sn(oct)_2_ catalyst in the ionic liquids (ILs); Teramoto and Nishio^36^ prepared cellulose diacetate-*graft*-poly(l-Lactide) copolymers with Sn(oct)_2_ in dimethyl sulfoxide (DMSO). However, the residual metals from catalyst are often found attached to the chain-end of the macromolecular products, thus may affect the quality of the products and narrow their applications in biomedical materials, food packaging or electronic^37–39^.

Following the first report of the use of 4-dimethylaminopyridine (DMAP) as an organic catalyst for ri﻿ng-opening polymerization (ROP) of lactide, a robust catalytic system were investigated for activity in ROP of cyclic esters, like acids^39, 40^, N-heterocyclic carbenes (NHC)^41^, amidines^42, 43^, guanidines^44^ and so on. Organocatalysis is a versatile strategy for ROP and provides a powerful alternative to the use of more traditional metallo-organic catalysts^39–41, 45, 46^. Many organic catalysts are simple, commercially available molecules that are typically easy to remove from the resultant polymers by simple washing or trapping in resin beads^44^. Previous studies about organic catalysts mediated ROP were mainly conducted on cellulose at high temperature^31, 39, 40^. However, the high temperature may lead to the degradation of the initial materials, resulting in the copolymers with low graft efficiency^34, 47^. Recently, 1,8-Diazabicyclo[5.4.0]undec-7-ene (DBU) has been applied as a competent nucleophilic catalyst for ROP by a number of researchers^48–50^. Advantageously, DBU mediated ROP is efficient under mild conditions^38, 42^.

Herein, we extend the study of ROGP of l-LA onto xylan-type hemicelluloses with the organic catalyst DBU to improve the graft efficiency under mild conditions. The physicochemical properties of the xylan derivatives were characterized by gel permeation chromatography (GPC), FT-IR, ^1^H-NMR, ^13^C-NMR, ^1^H-^1^H COSY, ^1^H-^13^C HSQC, ^1^H-^13^C HMBC, thermogravimetric analysis (TGA/DTG), differential scanning calorimetry (DSC) and X-ray diffraction (XRD).

## Results and Discussion

### The effects of reaction conditions on the ROGP of l-LA with xylan

**Figure 1:**
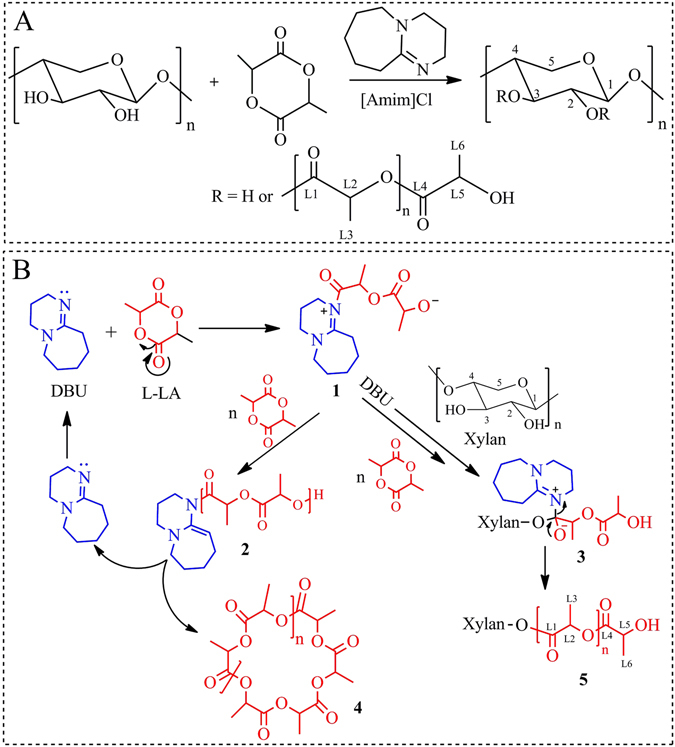
The grafting copolymerization of PLA onto xylan in [Amim]Cl with organic catalyst DBU (**A**) and the possible mechanism (**B**).

Organocatalysts such as DBU have been widely applied for ROP, and are considered as practical alternatives to organometallic and enzymatic catalysts due to their efficiency, scope and sustainability^42^. A homogeneous ROGP reaction of l-LA onto xylan backbone with the DBU catalyst was performed as shown in Fig. 1A. The proposed reaction mechanism is presented in Fig. 1B. As shown in Fig. 1B, grafting and self-polymerization of l-LA occurred simultaneously. Attack of the nucleophilic nitrogen of DBU on the carbon of the acetyl group in l-LA generated zwitterion **1**, analogous to the mechanisms proposed for the activation of acyl halides and dialkyl carbonates with DBU^49^. The acylated amidinium zwitterion **1** could react via several pathways^42, 51^. Zwitterion **1** could experience ring-closure and release a cyclic polylactide **4** and DBU. Alternatively, in the presence of excess DBU and hydroxyl groups in xylan as initiator, zwitterion **1** could undergo chain growth to generate xylan-*g*-PLA copolymers **5**.

Xylan-*g*-PLA copolymers with various graft lengths were synthesized by adjusting the reaction time, reaction temperature, catalyst concentration and molar ratio of monomer-to-AXU (anhydroxylose units in xylan). The experimental results are presented in Table 1. Grafting of PLA onto xylan at 50 °C with the DBU catalyst produced more and longer grafted side chains compared to the literature^34^. This result illustrated that the graft efficiency is sensitive to the catalyst and reaction conditions, particularly temperature. Dong *et al*.^23^ and Guo *et al*.^35^ observed a low graft efficiency for the synthesis of cellulose-*g*-PLA copolymers in ILs using the Sn(oct)_2_ catalyst at 130 and 110 °C, with a maximum degree of polymerization (DP) of 1.70 and 2.39, respectively. Yan *et al*.^34^ prepared thermoplastic cellulose-*g*-PLA copolymers by replacing Sn(oct)_2_ with an organic catalyst DMAP at 80 °C, and observed a dramatic improvement in the graft efficiency, with a maximum DP of 4.48. However, the high reaction temperatures used in these previous studies may have resulted in transesterification reactions^52^. In the present study, the grafting reaction was conducted at 50 °C, and the maximum DP was 5.51. In general, these results indicated that high graft efficiency can be achieved at 50 °C, probably due to the decreased degradation of the initial materials, the limited transesterification reactions and the practical advantages of DBU for catalyzing the ROGP of l-LA^38, 42, 47^.

**Table 1:** Compositional reaction parameters of xylan-*g*-PLA copolymers synthesized under different reaction conditions.

Sample	Temp. (°C)	Time (h)	l-LA/AXU (mol/mol)	DBU (wt)	DS	DP	MS	W_PLA_ (%)
1	110	1	8:1	2%	0.25	1.85	0.46	20.06
2	110	3	8:1	2%	0.47	2.51	1.18	39.16
3	110	6	8:1	2%	0.41	1.39	0.57	23.72
4	110	12	8:1	2%	0.24	1.36	0.33	15.25
5	110	24	8:1	2%	0.21	1.32	0.28	13.20
6	ambient	12	8:1	2%	0.06	1.76	0.11	5.67
7	40	12	8:1	2%	0.37	1.94	0.72	28.20
8	50	12	8:1	2%	0.41	2.65	1.09	37.29
9	70	12	8:1	2%	0.37	2.26	0.84	31.42
10	90	12	8:1	2%	0.31	2.07	0.64	25.88
11	130	12	8:1	2%	0.23	1.31	0.30	14.06
12	110	12	8:1	0.5%	0.13	1.18	0.15	7.56
13	110	12	8:1	1%	0.17	1.27	0.22	10.71
14	110	12	8:1	1.5%	0.21	1.33	0.28	13.25
15	110	12	8:1	4%	0.19	1.07	0.20	9.84
16	110	12	20:1	4%	0.25	1.94	0.49	21.09
17	50	12	8:1	0.5%	0.28	2.99	0.84	31.42
18	50	12	8:1	1%	0.33	3.37	1.11	37.71
19	50	12	8:1	1.5%	0.38	3.43	1.30	41.49
20	50	12	8:1	4%	0.45	4.06	1.83	49.95
21	50	12	20:1	4%	0.58	5.51	3.19	63.50
22	50	12	2:1	2%	0.15	2.07	0.31	14.46
23	50	12	4:1	2%	0.27	2.43	0.66	26.47
24	50	12	12:1	2%	0.45	4.09	1.84	50.09
25	50	12	20:1	2%	0.47	4.68	2.19	54.43

(DS, the degree of substitution of xylan-*g*-PLA copolymers, calculated by ^1^H-NMR; DP, the degree of polymerization of PLA side chains, calculated by ^1^H-NMR; MS, the molar substitution of xylan-*g*-PLA copolymers, calculated by ^1^H-NMR; and W_PLA_, the weight content of PLA side chains, calculated by ^1^H-NMR).

### FT-IR studies of the unmodified xylan and xylan-*g*-PLA copolymer

The unmodified xylan and xylan-*g*-PLA copolymer sample 21 (DS = 0.58) were characterized by FT-IR spectroscopy (see Supplementary Fig. S1). The characteristic absorbances at 3423, 2908, 1736, 1638, 1383, 1044 and 896 cm^−1^ were assigned to xylan backbone^9^. The FT-IR spectrum of xylan-*g*-PLA copolymer sample 21 revealed an intense absorption peak from the carbonyl group (C=O) at 1755 cm^−1^, confirming the presence of the PLA polymer. The intensity of the band at 3480 cm^−1^ for O-H stretching significantly decreased due to the decrease of hydroxyl groups in xylan. The O-H stretching vibration was shifted to a higher wave-number domain, probably due to the decreased hydrogen bonding force in xylan after modification. The absorption peaks at 2993 and 2945 cm^−1^ were attributed to -CH_3_ and -CH_2_- groups. The absorption bands at 1271, 1191 and 1091 cm^−1^ represented the backbone ester group of PLA.

### GPC analysis of the xylan-*g*-PLA copolymer

Xylan-*g*-PLA copolymer sample 21 was also characterized by GPC (see Supplementary Fig. S2). The single elution peak indicated the complete removal of PLA homopolymer. The GPC results (*M*_n_ = 81,200 g mol^−1^, *M*_w_ = 92,500 g mol^−1^) further confirmed the successful grafting of PLA onto xylan. More importantly, the narrow dispersity (PDI = 1.14) of the copolymer will facilitate the expansion of the application of xylan derivatives.

### 1D- and 2D-NMR analysis of the unmodified xylan and xylan-*g*-PLA copolymer

NMR is an effective characterization tool indispensable for analysis in the field of organic chemistry^53^. 1D (^1^H-NMR, ^13^C-NMR) and 2D (^1^H-^1^H COSY, ^1^H-^13^C HSQC, ^1^H-^13^C HMBC) NMR were performed to elucidate the structures of xylan and xylan-*g*-PLA copolymer sample 21 (DS = 0.58).

**Figure 2:**
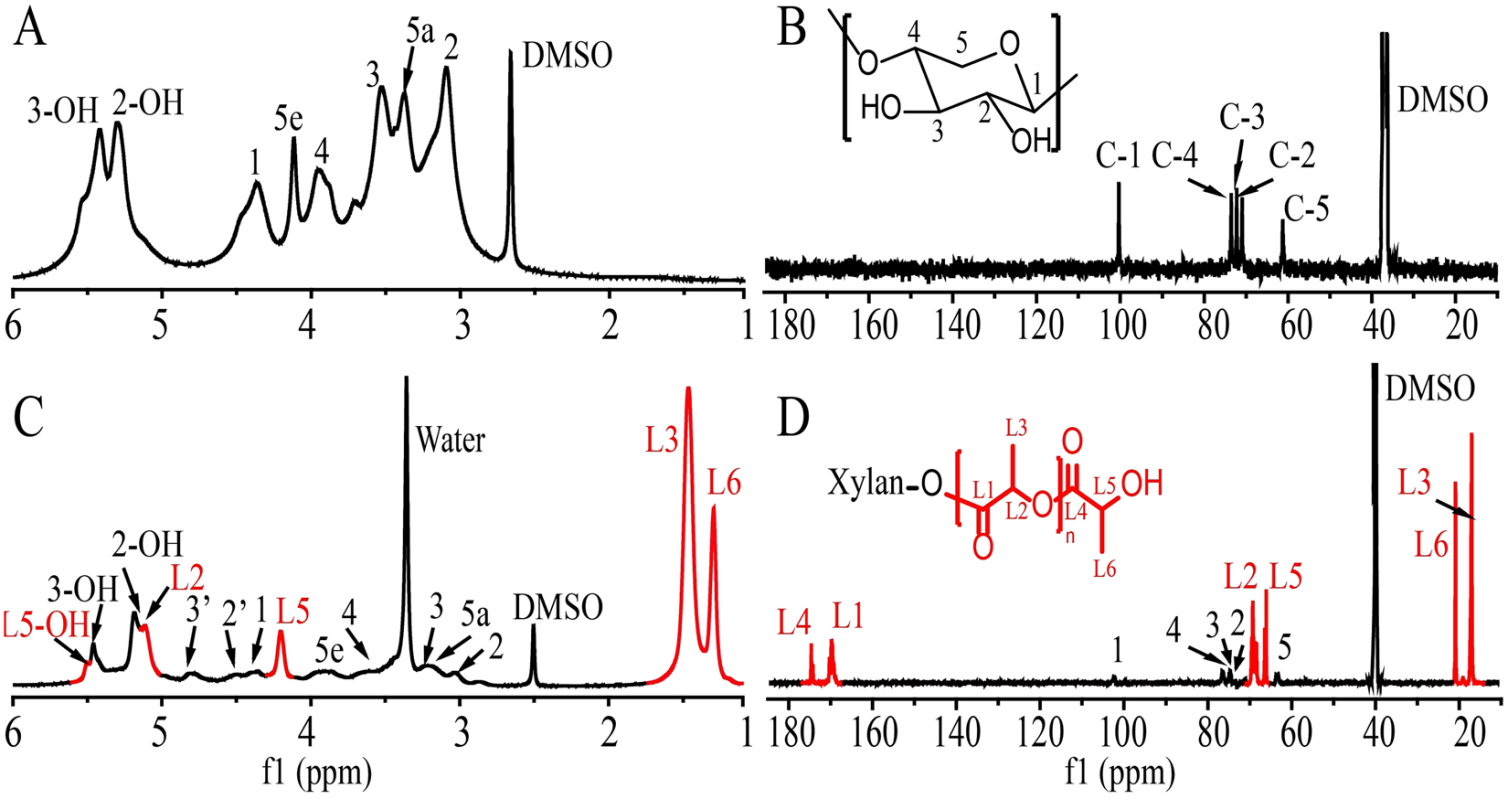
1D-NMR spectra of unmodified xylan (**A** for ^1^H-NMR, **B** for ^13^C-NMR) and xylan-*g*-PLA copolymer sample 21 (DS = 0.58, **C** for ^1^H-NMR, **D** for ^13^C-NMR).

Figure 2A presents the ^1^H-NMR spectrum of xylan. The *β*-(1–4)-linked d-Xylopyranose units were characterized by proton signals at 3.03, 3.32, 3.48, 3.92, 4.08 and 4.34 ppm, which were assigned to H-2, H-5_a_, H-3, H-4, H-5_e_ and H-1, respectively. The signals at 5.29 and 5.41 ppm were attributed to the protons from the hydroxyl groups in AXU^54^. In the ^1^H-NMR spectrum of xylan-*g*-PLA (Fig. 2C, sample 21, DS = 0.58), the proton signals of xylan were clearly evident. The signals at 1.29, 1.46, 4.21 and 5.12 ppm were assigned to the protons in the attached PLA side chains of L6, L3, L5 and L2, respectively. The hydroxyl group at the end of the PLA side chains (L5-OH) was responsible for the proton signal at 5.51 ppm. These results confirmed the successful graft polymerization of PLA on xylan. Figure 2B presents the ^13^C-NMR spectrum of xylan. The five strong signals at 102.1, 75.8, 74.5, 73.2 and 63.7 ppm were attributed to C-1, C-2, C-3, C-4 and C-5 of the AXU in xylan, respectively. In the ^13^C-NMR spectrum of xylan-*g*-PLA sample 21 (Fig. 2D, DS = 0.58), signals originated from the AXU of xylan were also detected. The signals at 17.1, 20.8, 65.9 and 69.2 ppm were assigned to L3, L6, L5 and L2 of the PLA side chains. In addition, the signals at 169.5 and 174.5 ppm were attributed to the carbonyl at the L1 and L4 positions. These carbon signals indicated the successful attachment of PLA onto the xylan backbone.

**Figure 3:**
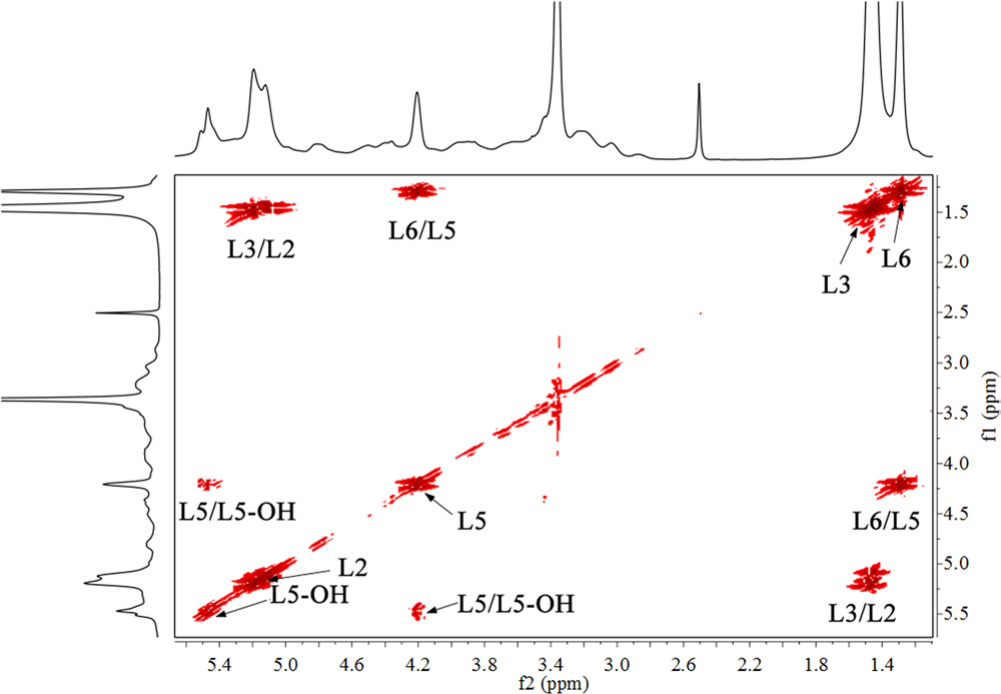
^1^H-^1^H COSY spectrum of xylan-*g*-PLA copolymer sample 21 (DS = 0.58).

To confirm the correct assignment of the proton signals of the attached PLA side chains, the ^1^H-^1^H COSY spectrum of xylan-*g*-PLA copolymer sample 21 (DS = 0.58) was acquired (Fig. 3). The spectrum is presented at a higher contour lever (the primary signals and cross-correlations in AXU are not shown) to clearly show the cross-correlations of the protons of the attached PLA side chains. Strong cross-correlations for L2/L3, L3/L2, L5/L5-OH and L5-OH/L5 of the PLA side chains were clearly observed. Moreover, the cross-correlations at δ_H_/δ_H_ of 1.29/4.21 and 4.21/1.29 ppm for L6/L5 and L5/L6 indicated the presence of the PLA repeating unit.

Based on the typical signals (L3 and L6 positions) from the PLA side chains and AXU in xylan (H-1 and H-4 positions), the molar substitution (MS), DS and DP of the xylan-*g*-PLA copolymers, and the weight content of PLA side chains (W_PLA_) (listed in Table 1) were estimated based on the integral area of the resonances for the corresponding protons, according to the following equations:

1 $${\rm{MS}}=\frac{({{\rm{I}}}_{{\rm{L}}3}+{{\rm{I}}}_{{\rm{L}}6})/3}{({{\rm{I}}}_{{\rm{H}}1}+{{\rm{I}}}_{{\rm{H}}4})/2}$$

2 $${\rm{DS}}=\frac{{{\rm{I}}}_{{\rm{L}}6}/3}{({{\rm{I}}}_{{\rm{H}}1}+{{\rm{I}}}_{{\rm{H}}4})/2}$$

3 $${\rm{DP}}=\frac{{\rm{MS}}}{{\rm{DS}}}=\frac{({{\rm{I}}}_{{\rm{L}}3}+{{\rm{I}}}_{{\rm{L}}6})/3}{{{\rm{I}}}_{{\rm{L}}6}/3}=1+\frac{{{\rm{I}}}_{{\rm{L}}3}}{{{\rm{I}}}_{{\rm{L}}6}}$$

4 $${{\rm{W}}}_{{\rm{PLA}}}=\frac{72{\rm{MS}}}{132+72{\rm{MS}}}\times 100 \% $$

where I_L3_ and I_L6_ are the integral areas of the lactyl-CH_3_ and terminal lactyl-CH_3_ protons respectively; I_H1_ and I_H4_ are the integral areas the of protons of the residual hydroxyls in xylan; 72 and 132 g mol^−1^ are the molecular weights of the l-LA monomer and xylan repeating unit, respectively.

**Figure 4:**
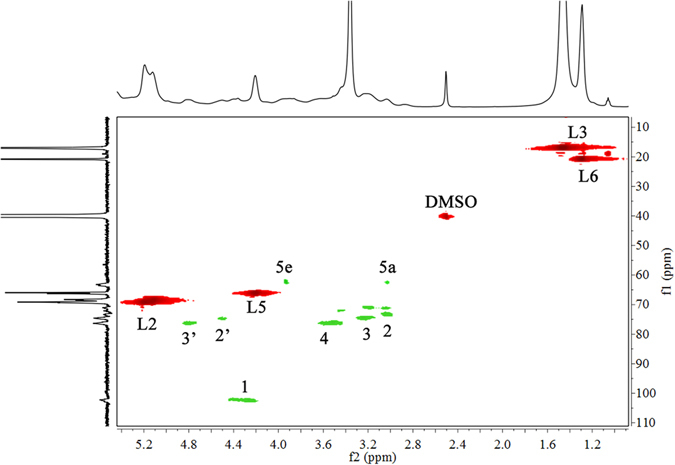
^1^H-^13^C HSQC spectrum of xylan-*g*-PLA copolymer sample 21 (DS = 0.58).

^1^H-^13^C HSQC provides detailed information on signal overlap in the ^1^H- and ^13^C-NMR spectra and can be applied for qualitative and quantitative analyses of chemical structures^4^. Figure 4 presents the ^1^H-^13^C HSQC spectrum of xylan-*g*-PLA copolymer sample 21 (DS = 0.58). The primary correlations from xylan and the PLA side chains are presented in Green and Red, respectively. The strong correlations at δ_C_/δ_H_ of 16.8/1.46, 20.7/1.28, 66.3/4.21 and 68.9/5.13 ppm were assigned to C_L3_/H_L3_, C_L6_/H_L6_, C_L5_/H_L5_ and C_L2_/H_L2_, respectively, indicating that the PLA side chains were successfully grafted onto xylan. Strong correlations of C_1_/H_1_, C_2_/H_2_, C_3_/H_3_, C_4_/H_4_, C_5e_/H_5e_ and C_5a_/H_5a_ in AXU were observed at δ_C_/δ_H_ of 101.9/4.35, 73.3/3.02, 75.1/3.24, 76.1/3.58, 63.1/3.92 and 63.1/3.17 ppm, respectively. Moreover, the correlations at δ_C_/δ_H_ of 73.3/4.49 and 75.1/4.80 ppm were assigned to the substituted C_2_/H_2_ and C_3_/H_3_ (2′ and 3′, respectively), indicating the ROGP of l-LA at C_2_ and C_3_ in the xylan backbone. According to the integrated resonances for substituted and unsubstituted C_2_/H_2_ and C_3_/H_3_, 46.88% and 53.12% of the PLA side chains were attached to C_2_ and C_3_, respectively.

**Figure 5:**
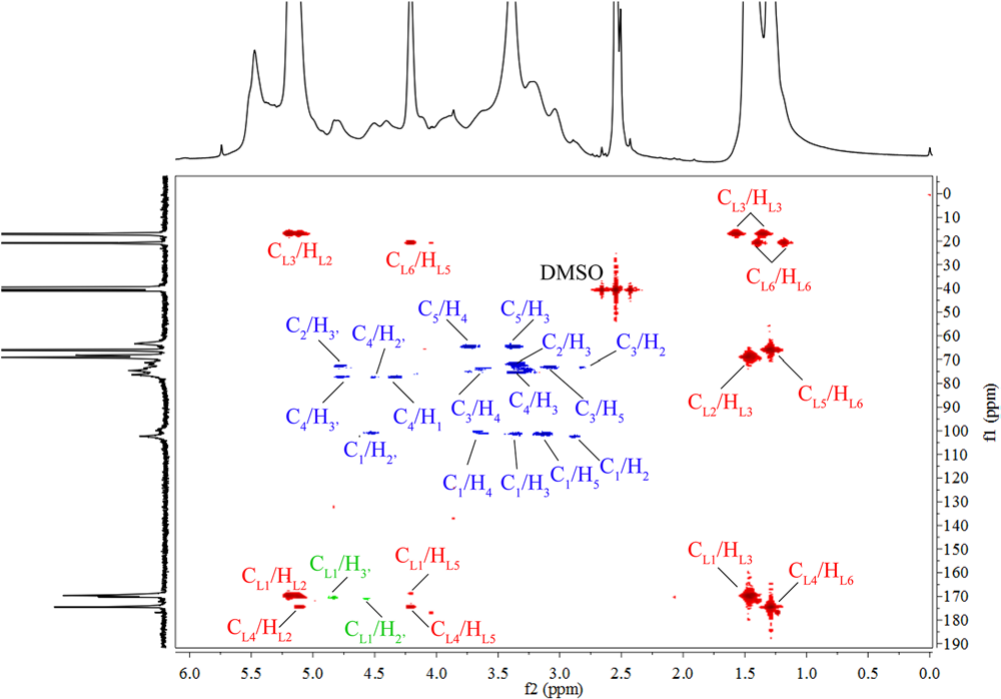
^1^H-^13^C HMBC spectrum of xylan-*g*-PLA copolymer sample 21 (DS = 0.58).

^1^H-^13^C HMBC is an effective tool to give correlations between carbons and protons that are separated by two, three, and sometimes in conjugated systems, four bonds. In theory, the positions of AXU attached with PLA side chains could be detected by ^1^H-^13^C HMBC. Herein, in order to further prove the attachment of PLA onto xylan backbone and the correct assignment of the primary signals of xylan-*g*-PLA copolymers, the ^1^H-^13^C HMBC spectrum of sample 21 (DS = 0.58) is presented in Fig. 5. To better understand the spectrum, the primary correlations from xylan backbone, PLA side chains and cross-correlations between xylan and PLA are shown in Blue, Red and Green. Expectedly, the Green correlations at δ_C_/δ_H_ of 170.2/4.82 ppm (C_L1_/H_3′_) and 170.2/4.49 ppm (C_L1_/H_2′_) are assigned to the cross-correlations between carbonyl carbon (C = O, L1 position) in PLA side chains and the protons at the substituted C_3_ and C_2_ (3′ and 2′ positions), respectively. These results confirmed the successful grafting of PLA side chains onto xylan backbone. Apparently, more PLA side chains were attached to C_3_ position than to C_2_ position, corresponding to the results from ^1^H-^13^C HSQC. The cross-correlation at δ_C_/δ_H_ of 175.3/5.11 ppm (C_L4_/H_L2_) illustrated the repeated PLA side chains. The other cross-correlation were detected in Red at δ_C_/δ_H_ of C_L1_/H_L2_ (170.2/5.11 ppm), C_L1_/H_L3_ (170.2/1.44 ppm), C_L1_/H_L5_ (169.9/4.18 ppm), C_L2_/H_L3_ (70.7/1.44 ppm), C_L3_/H_L2_ (17.5/5.11 ppm), C_L3_/H_L3_ (17.5/1.36 and 17.5/1.55 ppm), C_L4_/H_L5_ (175.3/4.18 ppm), C_L4_/H_L6_ (175.3/1.28 ppm), C_L5_/H_L6_ (65.8/1.28 ppm), C_L6_/H_L5_ (21.1/4.18 ppm) and C_L6_/H_L6_ (21.1/1.17 and 21.1/1.36 ppm), confirming the correct assignments of primary carbon and proton signals in PLA side chains. Moreover, the cross-correlations originating from AXU in xylan were observed in Blue at δ_C_/δ_H_ of 102.7/2.84 (C_1_/H_2_), 102.7/3.05 (C_1_/H_5_), 102.7/3.25 (C_1_/H_3_), 102.7/4.49 (C_1_/H_2′_), 76.8/3.25 (C_4_/H_3_), 76.8/4.49 (C_4_/H_2′_), 76.8/4.82 (C_4_/H_3′_), 74.9/3.66 (C_3_/H_4_), 74.9/3.05 (C_3_/H_5_), 74.9/2.84 (C_3_/H_2_), 71.9/3.25 (C_2_/H_3_), 71.9/4.84 (C_2_/H_3′_), 63.5/3.69 (C_5_/H_4_), and 63.5/3.25 ppm (C_5_/H_3_). The cross-correlations at δ_C_/δ_H_ of 102.7/3.66 (C_1_/H_4_) and 76.8/4.41 (C_4_/H_1_) ppm are associated with the linked xylose unit by *β*-1,4 linkage. The other cross-correlations from xylan were not detected under the selected contour level.

### Thermal analysis

**Figure 6:**
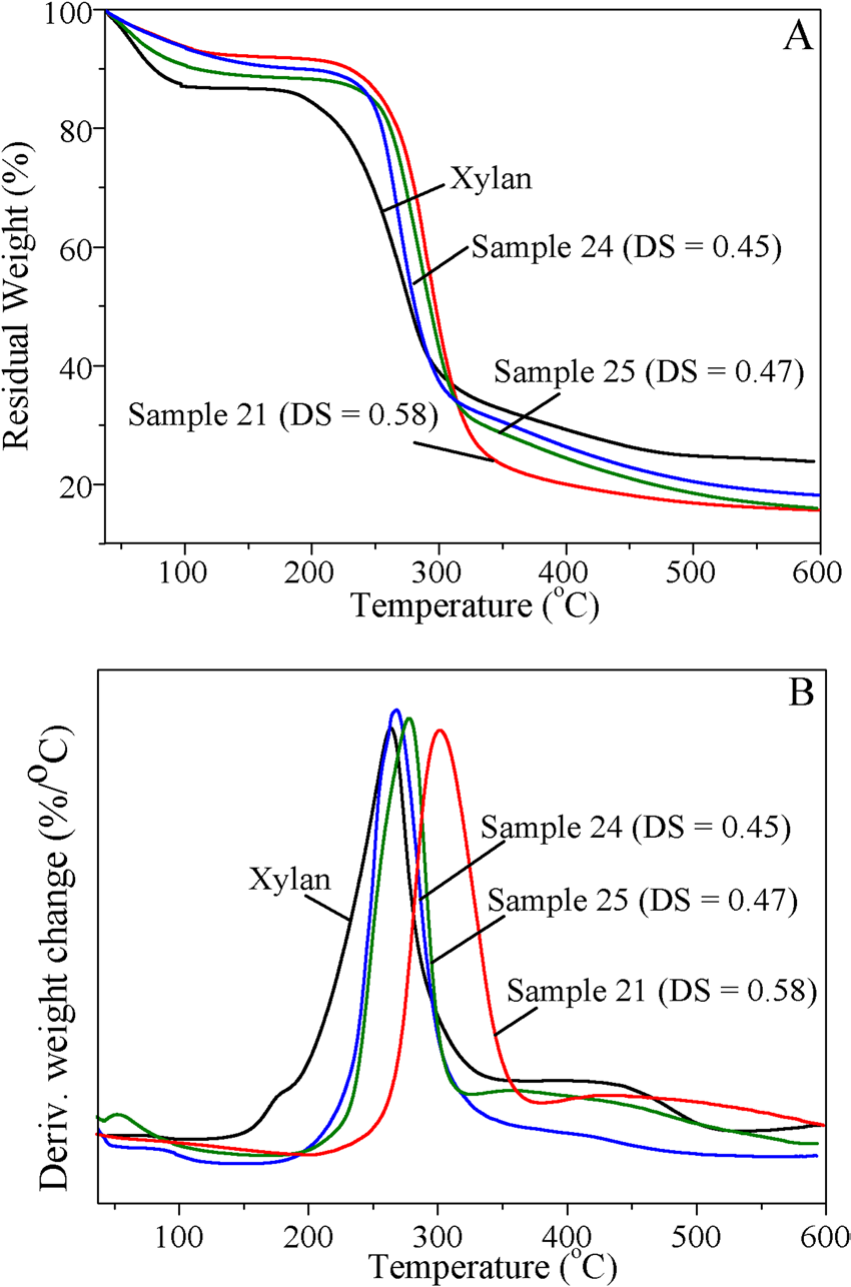
TGA (**A**) and DTG (**B**) curves of xylan and xylan-*g*-PLA copolymers samples 21 (DS = 0.58), 24 (DS = 0.45) and 25 (DS = 0.47).

The decomposition pattern and thermal stability of xylan and xylan-*g*-PLA copolymers with different DS were studied by TGA and DTG. As shown in Fig. 6A, the weight loss in the examined temperature range could be divided into three stages. The first drop in the curves was attributable to water loss, representing approximately 13% (xylan), 10% (sample 24), 11% (sample 25), and 8% (sample 21) of the initial weight, respectively, which was probably due to the increased hydrophobic nature of the xylan derivatives after the grafting of PLA side chains^55^. Unmodified xylan began to decompose at 180 °C, whereas the xylan-*g*-PLA copolymers began to decompose at approximately 225 °C. At 50% weight loss, the decomposition temperatures of xylan and the grafted copolymers 24, 25, and 21 were 275 °C, 279 °C, 295 °C, and 300 °C, respectively. These results indicated that the thermal stability of xylan was enhanced by the increased DS of the xylan-*g*-PLA copolymers, in contrast to the decreased thermal properties of cellulose-*g*-PLA copolymers with increased DS^14, 35^. These differences are probably due to the diversities of structure between cellulose and xylan. Cellulose in its natural state has a crystalline structure, resulting in a high onset decomposition temperature of approximately 300 °C^14^. Grafting with PLA side chains may disrupt the crystalline structure of cellulose, leading to the decreased thermal stability of the cellulose-*g*-PLA copolymers. At 600 °C, the pyrolysis residues of xylan and samples 24, 25 and 21 were 24%, 18%, 15% and 15%, respectively, indicating that the inorganic salts content of the samples decreased after modification^13^.

DTG_max_ indicates the maximum degradation rate and can be used to compare the thermal stability between samples. In the DTG curves (Fig. 6B), xylan and samples 24, 25 and 21 exhibited DTG_max_ at approximately 270, 275, 280 and 300 °C, respectively. Thus, the degradation range of xylan was decreased after grafting of the PLA side chains, indicating that chemical modification led to the increased thermal stability. However, the graft copolymers decomposed in one step, in contrast to the thermal degradation of cellulose-*g*-PLA copolymers, which proceeded in two steps for the decomposition of PLA and cellulose^14^. The single-step decomposition in the present study was probably due to the low MS of the copolymers, similar to the results observed by Yan *et al*.^34^.

### DSC analysis

**Figure 7:**
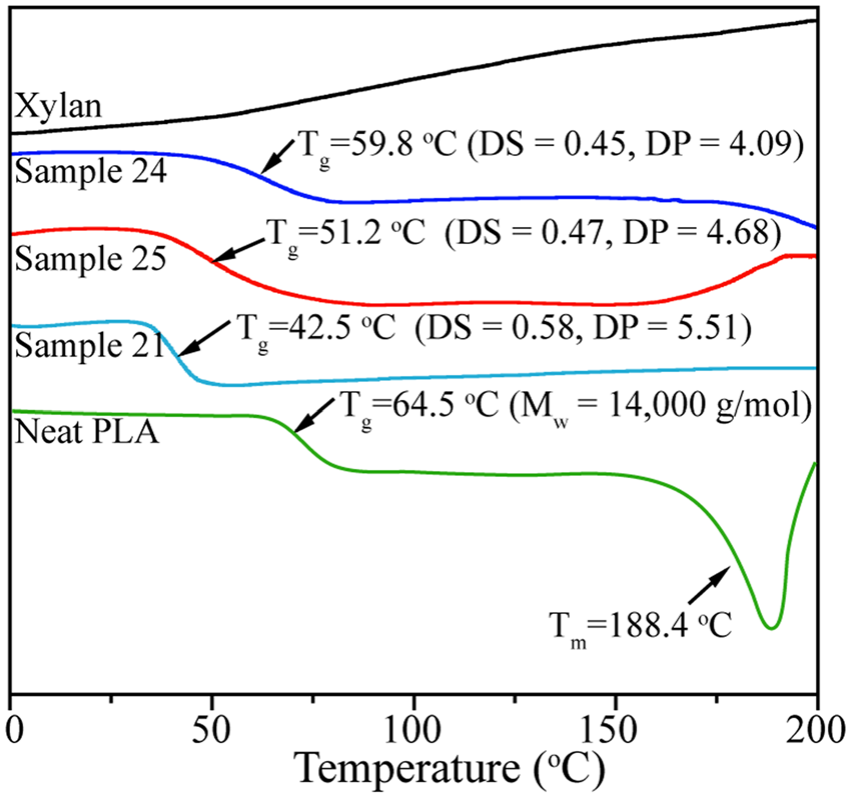
DSC curves of xylan, neat PLA (*M*_w_ = 14,000 g mol^−1^) and xylan-*g*-PLA copolymers samples 21 (DS = 0.58), 24 (DS = 0.45) and 25 (DS = 0.47).

Thermograms of unmodified xylan, xylan-*g*-PLA copolymers and commercial neat PLA (*M*_w_ = 14,000 g mol^−1^) on second heating are presented in Fig. 7. No melting temperature (*T*_m_) or glass transition temperature (*T*_g_) was observed in the curve of unmodified xylan, indicating that the xylan was amorphous^5, 55^. The neat PLA exhibited a *T*_m_ of approximately 188.4 °C and a *T*_g_ of 64.5 °C, consistent with the literature^11, 56^. For the xylan-*g*-PLA copolymers, each curve exhibited a clear *T*_g_. As MS increasing from 1.84 to 3.19 (DS from 0.45 to 0.58), *T*_g_ decreased from 59.8 °C to 42.5 °C, indicating that the relatively long grafted PLA side chains increased the intermolecular distance and chain mobility, thus playing an effective role as an internal plasticizer of xylan^11, 57^. The tunable *T*_g_ values of the xylan-*g*-PLA copolymers indicated the probability of subsequent melt-processing of the materials under mild conditions into biobased plastic products with desirable thermal tolerance. No melting peaks were observed in the DSC curves of the copolymers, indicating that the copolymers were amorphous and consistent with the results of the XRD measurements. Previously reported cellulose reinforced PLA biocomposites were also amorphous^34, 56, 57^.

### XRD analysis

X-ray diffractograms of unmodified xylan, neat PLA (*M*_w_ = 14,000 g mol^−1^) and xylan-*g*-PLA copolymer sample 21 (DS = 0.58) are investigated (Supplementary Fig. S3). Unmodified xylan exhibited a broad diffraction peak at 2θ = 19.2°, indicating that xylan was amorphous. The neat PLA was semicrystalline, with two main diffraction peaks at 2θ = 16.6° and 18.9°. In the XRD curve of xylan-*g*-PLA copolymer sample 21, only one disperse broad peak around 2θ = 19.5° was observed, indicating that the xylan-*g*-PLA copolymers were amorphous. This probably due to the grafted PLA side chains were not sufficiently long to form a new crystalline structure, in agreement with the DSC results. Consequently, the PLA-reinforced biocomposites were indeed non-crystalline, as previously reported^23, 57^.

## Conclusion

In the present study, xylan-*g*-PLA copolymers with high graft efficiency were successfully synthesized in [Amim]Cl under mild conditions. The hydroxyl groups on xylan acted as initiators in ROGP of l-LA, and DBU was applied as an organic catalyst to enhance the nucleophilicity of the initiator and propagate the PLA chain. By varying the reaction conditions, the DS and DP of the obtained copolymers could be selectively tuned, resulting in a maximum DS of 0.58 and DP of 5.51 at 50 °C. GPC, FT-IR and NMR confirmed the successful ROGP of l-LA with xylan. ^1^H-^13^C HSQC analysis of the structure of the xylan-*g*-PLA copolymers indicated that 46.88% and 53.12% of the PLA side chains were attached to C_2_ and C_3_ of xylan, respectively. The thermochemical properties of the copolymers indicated that the modification transformed xylan to a thermoplastic material with a tunable glass-transition temperature (*T*_g_) from 42 °C to 60 °C. Further studies will be focused on the application of xylan-*g*-PLA copolymers.

## Experimental

### Materials

Xylan with a xylose content of greater than 90% (isolated from beech wood) was purchased from Sigma-Aldrich Co., LLC (Shanghai, China). The average molar mass of xylan was 58,000 g mol^−1^. l-LA and neat PLA (*M*_w_ = 14,000 g mol^−1^) with a purity of 99.5% were purchased from Sigma-Aldrich Co., LLC (Shanghai, China). DBU with a purity of 99% was purchased from Aladdin Reagent Co. (Shanghai, China). [Amim]Cl with a purity of 99% was supplied by Cheng-Jie Chemical Co., Ltd. (Shanghai, China) and was dried under vacuum for 48 h at 70 °C before use. All other chemicals were of analytical reagent grade and were used directly without further purification.

### Synthesis of xylan-*g*-PLA copolymer

A typical procedure for the grafting of PLA onto xylan was as follows. Dry xylan (0.11 g, 1.67 mmol) was dissolved in [Amim]Cl (10 g) at 80 °C in a sealed 25-mL three-neck reaction flask. The xylan solution was treated with three cycles of vacuum and nitrogen (N_2_) under vigorous stirring to remove moisture. After approximately 1 h, l-LA was added to the solution, and the same procedure described above was followed to remove moisture. Then, DBU as a catalyst was slowly added to the solution, and the reaction was performed at an appropriate temperature and time under a N_2_ atmosphere with vigorous stirring. After cooling to room temperature, the xylan-*g*-PLA copolymers were isolated by precipitation in deionized water and subsequently purified by dialysis against water to remove [Amim]Cl, catalyst and unreacted monomer. The copolymers were then washed by Soxhlet extraction with dichloromethane for 24 h to completely remove any free homopolymer, and dried under vacuum for 48 h at 70 °C.

### Characterization

GPC was performed using a Waters model 1515 (USA) equipped with a 2414 differential detector and Styragel HR3 and Styragel HR4 columns. The measurement was performed at 35 °C. N,N-dimethylform﻿amide (DMF) was used as the mobile phase (0.6 mL/min). The run time and volume were 50.0 min and 50.00 μL, respectively.

FT-IR spectra of the unmodified xylan and xylan-*g*-PLA copolymers were obtained in the range of 4000–400 cm^−1^ using a Bruker spectrophotometer (Tensor 27, Germany) in KBr disc containing 1% (w/w) of finely ground sample.

^1^H-NMR, ^1^H-^1^H COSY, ^13^C-NMR, ^1^H-^13^C HSQC, ^1^H-^13^C HMBC spectra were recorded from 40 mg samples in 0.5 mL of DMSO-*d*_*6*_ on a Bruker AVIII 600 M spectrometer (Germany) with a 5 mm multinuclear probe (see Supplementary Experimental Details).

TGA/DTG was performed using a TGA Q500 thermogravimetric analyzer (TA, USA). The sample (9 to 11 mg) was heated from 30 °C to 600 °C at a heating rate of 10 °C/min and an air flow rate of 25 mL/min.

DSC (Q200, USA) was performed to assess the *T*_g_. The specimens (5 to 7 mg) were transferred into the pan used for DSC measurements. For the successive heating/cooling cycling measurements, the following thermal procedure was used: 10 °C/min ramp from ambient temperature to 100 °C, isotherm at 100 °C for 5 min, cool to 0 °C, isotherm at 0 °C for 5 min, 10 °C/min ramp from 0 °C to 200 °C and cool to ambient temperature naturally.

XRD was performed using a D/max-III A X-ray diffractometer (Japan) with nickel-filtered Cu Kα radiation (40 kV, 40 mA). Data were measured in a range of 2θ = 5 to 40° with a step size of 0.04° and time per step of 0.2 s at room temperature.

## Electronic supplementary material

Synthesis of Thermoplastic Xylan-Lactide Copolymer with Amidine-Mediated Organocatalyst in Ionic Liquid
