# Supplementary material for: Synthesis of Thermoplastic Xylan-Lactide Copolymer with Amidine-Mediated Organocatalyst in Ionic Liquid
Source: Sci Rep. 2017 Apr 3;7:551. doi: 10.1038/s41598-017-00464-6 (PMC5428448; doi:10.1038/s41598-017-00464-6)
Supplement: Supplementary file 1 — Synthesis of Thermoplastic Xylan-Lactide Copolymer with Amidine-Mediated Organocatalyst in Ionic Liquid [file 41598_2017_464_MOESM1_ESM.docx]

Supplementary Information

Synthesis of Thermoplastic Xylan-Lactide Copolymer with Amidine-Mediated Organocatalyst in Ionic Liquid

Xueqin Zhang^1^, Huihui Wang^1^, Chuanfu Liu^1,*^, Aiping Zhang^2^, and Junli Ren^1^

*1 State Key Laboratory of Pulp and Paper Engineering, South China University of Technology, Guangzhou 510640, China*

*2 College of Materials and Energy, Guangdong Key Laboratory for Innovative Development and Utilization of Forest Plant Germplasm, South China Agricultural University, Guangzhou 510642, P. R. China*

*corresponding author: [chfliu@scut.edu.cn](mailto:chfliu@scut.edu.cn)

**Experimental Details**

For the ^1^H-NMR analysis, the detailed collecting and processing parameters were listed as follows: number of scans, 16; receiver gain, 14; acquisition time, 2.7263 s; relaxation delay, 1.0 s; pulse width, 11.0 s; spectrometer frequency, 600.17 MHz; and spectral width, 12019.2 Hz. For the ^1^H-^1^H COSY analysis, the detailed collecting and processing parameters were listed as follows: number of scans, 8; receiver gain, 447; acquisition time, 0.4588 s; relaxation delay, 2.0 s; pulse width, 9.0 s; spectrometer frequency, 400.13/400.13 MHz; and spectral width, 4000.0/4000.0 Hz. For the ^13^C-NMR analysis, the detailed collecting and processing parameters were listed as follows: number of scans, 6000; receiver gain, 187; acquisition time, 0.9088 s; relaxation delay, 2.0 s; pulse width, 12.0 s; spectrometer frequency, 150.91 MHz; and spectral width, 36057.7 Hz. For the ^1^H-^13^C HSQC analysis, the detailed collecting and processing parameters were listed as follows: number of scans, 28; receiver gain, 2050; acquisition time, 0.0639 s; relaxation delay, 2.0 s; pulse width, 8.5 s; spectrometer frequency, 400.13/100.61 MHz; and spectral width, 8012.8/20161.3 Hz. For the ^1^H-^13^C HMBC analysis, the detailed collecting and processing parameters were listed as follows: number of scans, 48; receiver gain, 187; acquisition time, 0.1434 s; relaxation delay, 1.4877 s; pulse width, 11.0 s; spectrometer frequency, 600.17/150.91 MHz; and spectral width, 7142.9.5/33557.0 Hz.

**
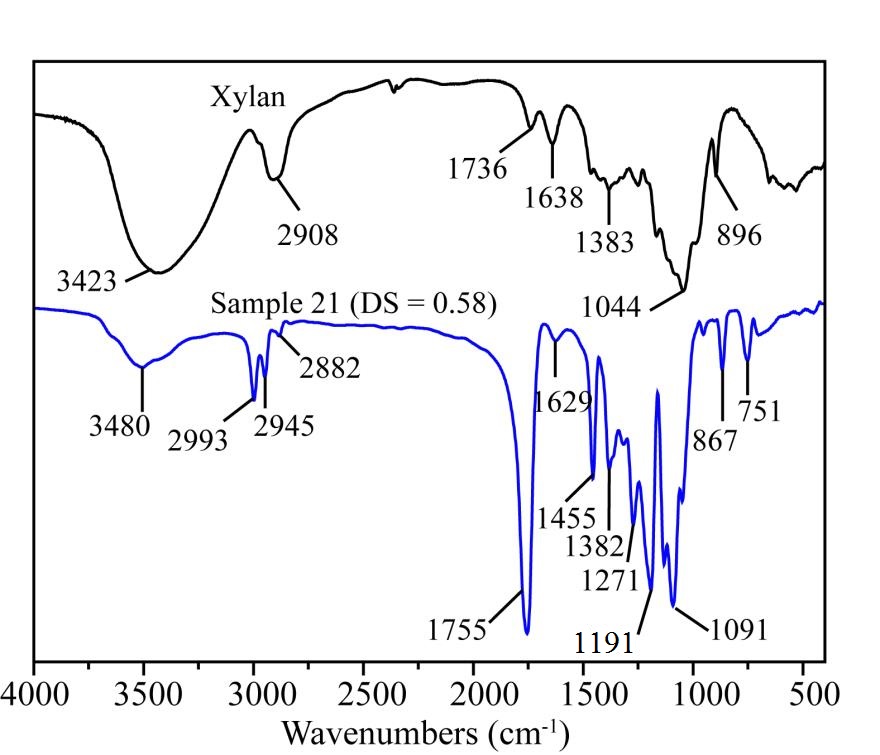
**

**Supplementary Figure S1.** FT-IR spectra of unmodified xylan and xylan-*g*-PLA copolymer sample 21 (DS = 0.58).


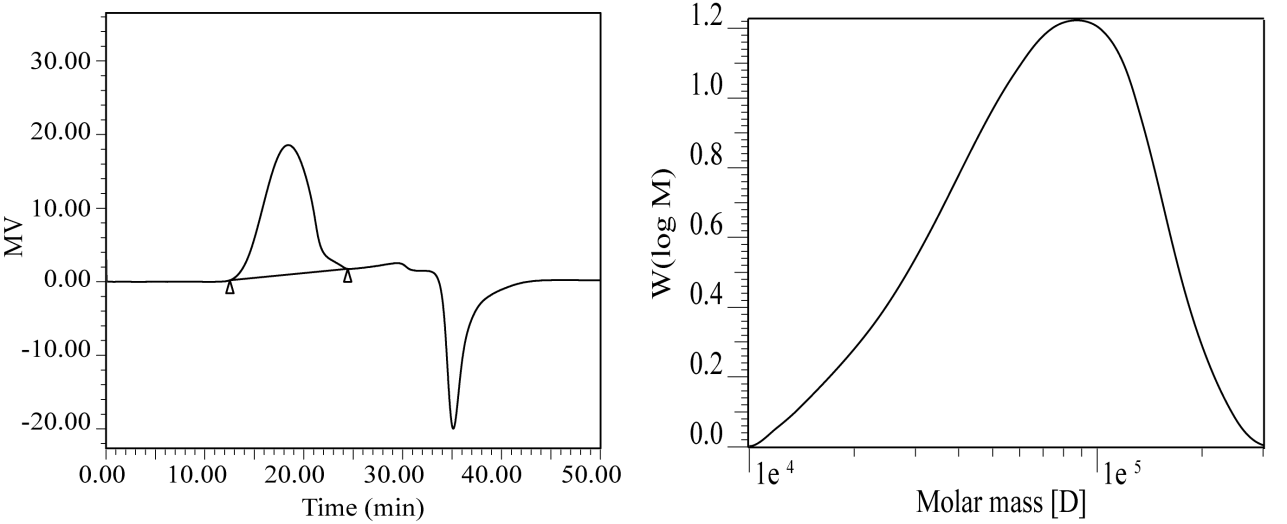


**Supplementary Figure S2.** GPC curves of xylan-*g*-PLA copolymer sample 21 (DS = 0.58).

**
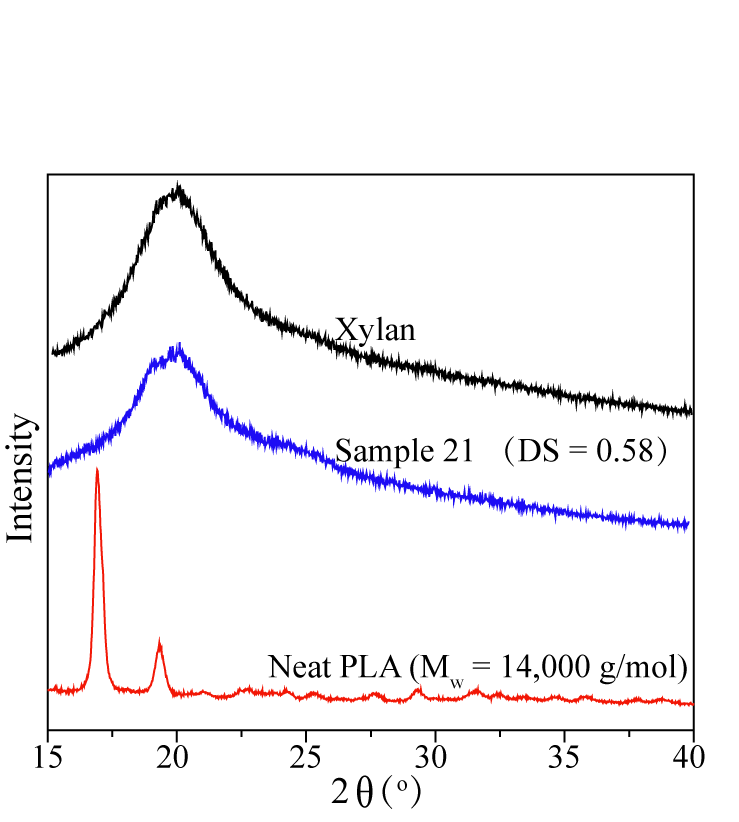
**

**Supplementary Figure S3** XRD curves of xylan, neat PLA (*M*_w_ = 14,000 g/mol) and xylan-*g*-PLA copolymer sample 21 (DS = 0.58).
